# Primacy of mouth over eyes to perceive audiovisual Mandarin lexical tones

**DOI:** 10.16910/jemr.16.4.4

**Published:** 2023-11-29

**Authors:** Biao Zeng, Guoxing Yu, Nabil Hasshim, Shanhu Hong

**Affiliations:** University of South Wales, Pontypridd, UK; University of Bristol, Bristol, UK; De Montfort University, Leicester, UK; Quanzhou Preschool Education College, Quanzhou, China

**Keywords:** lexical tone, eye movement, gaze, audiovisual speech, Chinese speakers, English speakers

## Abstract

The visual cues of lexical tones are more implicit and much less investigated than consonants and vowels, and it is still unclear what facial areas contribute to facial tones identification. This study investigated Chinese and English speakers’ eye movements when they were asked to identify audiovisual Mandarin lexical tones. The Chinese and English speakers were presented with an audiovisual clip of Mandarin monosyllables (for instance, /ă/, /à/, /ĭ/, /ì/) and were asked to identify whether the syllables were a dipping tone (/ă/, / ĭ/) or a falling tone (/ à/, /ì/). These audiovisual syllables were presented in clear, noisy and silent (absence of audio signal) conditions. An eye-tracker recorded the participants’ eye movements. Results showed that the participants gazed more at the mouth than the eyes. In addition, when acoustic conditions became adverse, both the Chinese and English speakers increased their gaze duration at the mouth rather than at the eyes. The findings suggested that the mouth is the primary area that listeners utilise in their perception of audiovisual lexical tones. The similar eye movements between the Chinese and English speakers imply that the mouth acts as a perceptual cue that provides articulatory information, as opposed to social and pragmatic information.

## Introduction

Speech communication in everyday life is, at least, bimodal communication. During face-to-face conversation, people integrate visual and auditory information automatically and, under some adverse conditions (e.g., noise, accent), visual cues can facilitate listener’s perception of sound (40; 23). Regarding visual cues in speech perception, Kim and Davis (19) distinguished between visual form and visual timing information. Visual form information includes the shape and movement of the mouth, lips and tongue and is known to help listeners identify spoken segments. On the other hand, visual timing information is derived from the peri-oral regions such as the head, neck and eyebrows as well as from global facial motions. These areas indicate the onset, offset and duration of spoken segments (36). Visual timing information also incorporates the cycling motion of opening and closing of the jaw, which provides rhythmic information regarding the syllables spoken (14; 26).

However, the visual cues of prosodic information are much more implicit than those of segmental consonants and vowels. Fisher (13) forged the concept of viseme to describe these cues and defined it as the smallest visible speech unit analogue to the phoneme. Chen and Massaro (7) defined visemes as articulatory manner and places (consonant) and mouth shapes (vowels). The visemic features of segmental consonants and vowels represent the articulatory gestures (places, e.g., bilabial /b/, manner e.g., fricative /v/), or mouth shapes, e.g., roundness /o/ or flatness /i/ in speech.

Currently most eye-tracking studies focus on segmental information. Intonation is a form of prosodic information which refers to the rise and fall of pitch over entire phrases and sentences. It conveys emotional, pragmatic, and social information, e.g., questioning, doubting and satire. The relevant building blocks of intonation (tonal events, i.e., pitch accents, boundary tones) are carried over by vowels. Intonation information may be present in a one syllable or in a multiword utterance. Several studies have reported the role of upper facial cues in this form of prosodic information. The upper facial cues can facilitate the listener’s ability to identify intonation through head movements (31; 20; 10, 11) and eyebrow movements (6; 21; 18; 8).

Lexical tone is another form of prosodic information. 70% of languages in the world are tonal languages and lexical tones widely exist in many Asian and African languages (46). For instance, Mandarin has four different tones: mā (Tone 1, high, 55(the numbers represent tone height), mother), má (Tone 2, rising, 35, hemp), mă (Tone 3, dipping, 214, horse), and mà (Tone 4, falling, 51, scold). Similar to intonation, lexical tone is determined by the fundamental frequency (F0) height and contour. However, functional and acoustic differences exist. Functionally, lexical tones convey semantic information and distinguish different words. Intonation indicates pragmatic and social information, e.g., attitude and emotion, which could incorporate in many visual gestures, facial expression and even body movement. Acoustically, although both intonation and lexical tone primarily involve pitch variation, intonation may be present in one syllable or a multiword utterance. Lexical tone is produced using vowels (15, 16; 22). Therefore, lexical tone does not lead to the production of detailed articulatory gestures involving upper face and head, when compared to intonation.

In terms of visual cues, lexical tone cues are far less researched than those relating to intonation. Preliminary studies of visual benefit effect have reported that adding visual information could improve perception of lexical tones (29; 30; 4; 7; 42). However, vibrations of vocal cords are responsible for the production of lexical tones; and these rarely result in visual cues being presented via the shape and movement of the speaker’s mouth. Therefore, it is unclear what specific facial areas contribute to the identification of lexical tones.

Both intonation and lexical tone fall under the scope of pitch frequency and are produced by the vocal cords. Therefore, the visual cues contributing to lexical tone may be similar to those involved in intonation, such as head movements and upper facial cues. It is probable that the eye area would be more helpful in identifying lexical tones than the mouth. This is supported by Swerts and Krahmer (37), who reported that upper facial areas had stronger cue values than the lower areas.

Alternatively, intonation occurs across a relatively long utterance compared to lexical tone. Thus, the length of utterance gives listeners more opportunities to attend to the visual cues that are derived from upper facial areas. Whereas, for a short lexical tone, visual information might be primarily extracted from the mouth area, which offers phonetic information regarding the syllabic length. Therefore, it may be useful to analyse listener’s gaze in order to identify which areas of the face are utilised as a cue in the perception of lexical tones.

Gaze allocation might also be influenced by other factors, such as the acoustic environment. When presenting Japanese and English speech to participants, Vatikiotis-Bateson et al. (41) found that participants gazed more at the mouth when noise levels increased. Yi, Wong and Eizenman (45) replicated these results and confirmed that whilst the mouth and eye areas were the two primary regions of interest in audiovisual speech, the listeners directed gaze more towards the mouth when the acoustic signal became weaker.

Previous studies of cross-language comparison have revealed differences in the use of visual information between participants from different linguistic and cultural backgrounds. This is exemplified by audiovisual speech perception research involving the McGurk effect. McGurk and MacDonald (28) dubbed a sound of /ba/ on to lip movement for /ga/. A hearing illusion of /da/ was observed and named as fusion. The reverse dubbing process (auditory /ga/ and visual /ba/) might produce the other illusion of combinations (a hearing illusion of /bagba/ or /gaba/). This is the McGurk effect, and it has been widely cited as a paradigmatic probe of multisensory integration across modalities (1).

Sekiyama and Tohkura (34) found the McGurk effect was significantly more pronounced in American participants compared to Japanese participants when they processed Japanese and English syllables. Another experiment from Sekiyama (33) showed that compared with American participants, Chinese participants also showed a weak McGurk effect when they processed syllables like /ba/, /pa/, /ma/ and so on, similar to Japanese participants. Sekiyama (33) attributed this result to two aspects: one was a cultural factor, both Chinese and Japanese people tend to avoid looking at each other’s face when communicating, which leads them to be poorer at using visual information for speech recognition; the other was a language factor as Chinese Mandarin is a tonal language, its acoustic characteristics leave listeners more dependent on auditory information during speech recognition. It can be seen from the above research that the use of visual information for audiovisual speech perception varies among different language speakers: English speakers are much more affected by visual information than Japanese and Chinese speakers.

Nevertheless, using a large sample of 162 Chinese participants and 145 American participants, Magnotti et al. (27) reported the use of visual information to be at a similar frequency between the two groups: the McGurk effect ratio between Chinese and American participants was 48% and 44%, respectively, which further disputed the idea of a cultural difference explaining previous findings produced by Sekiyama and others (34; 33; 17).

The present study used an eye-tracker to compare the eye movements of Chinese and English speakers who were asked to identify Mandarin lexical tone in a two-alternative forced-choice (2FAC) task. The 2FAC task requires participants to identify lexical tones at the perceptual level. As non-native tonal language speakers, English participants have no lexical tone representations in their mental lexicon and would process lexical tones without accessing semantic information. Both Chinese and English speakers would rely on perceptual cues differing from those involved in intonation and extraction of pragmatic or emotion information, for instance, eyebrow movements.

Eye-tracking studies have implicated the mouth and eyes as two primary regions involved in the processing of audiovisual speech, with the majority of studies supporting the primacy of mouth (41; 24; 32; 45; 25; 9). Therefore, two hypotheses were investigated. Firstly, we assume the primacy of mouth over eyes, which indicates the participants will gaze at the mouth area longer than eye areas when processing lexical tones. This is likely to be especially true under adverse listening conditions. Secondly, we assume no differences in eye movement patterns will exist between the groups of native Chinese and English speakers.

## Methods

### Participants

Twenty-one participants (mean age = 29.2 years, age range = 19.0 - 43.2) took part in the study. 11 (7 females, 4 males) were native Mandarin speakers while the rest (7 females, 3 males) were native English speakers who did not speak any tonal language. Participants were paid £8/hour for their participation. were recruited from the Bournemouth University student community as participants in the current study. They all reported normal or corrected-to-normal visual acuity and no hearing impairment. The experimental protocol was approved by the Research Ethics Panel of Bournemouth University in accordance with the Declaration of Helsinki. Informed consent was obtained from each participant before the experiment took place.

### Design and Materials

**Figure 1.**
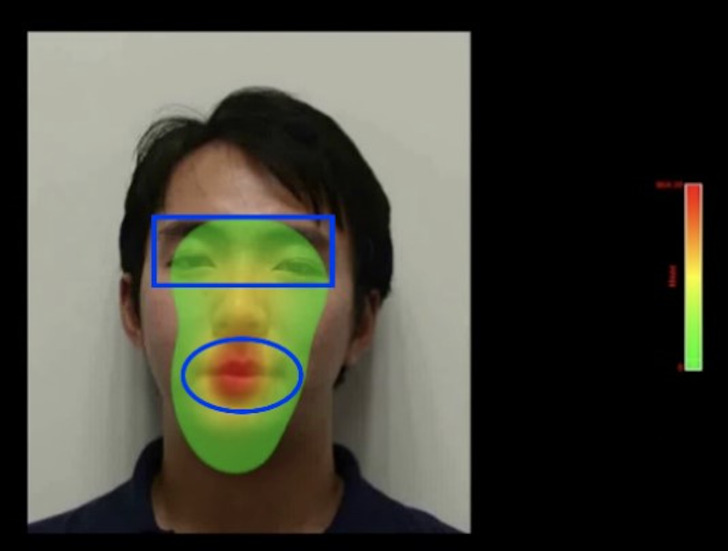
Face in the left side of a screen and takes 2/3 of this full screen. Red colour indicates longer gaze duration and the green colour shorter gaze duration.

Video and audio clips of two different speakers were used throughout the experiment. During the recording, the speakers kept their head still to avoid supplying any additional head movement cue. On each trial, a video of one speaker was played either to the left or right side of the screen, so that the initial gaze at the center of the screen (a central fixation cross) was not on any part of the speaker’s face. Each speaker kept their head still and pronounced each syllable. The video displayed the speaker’s full face from above the neck (see in Figure 1) and took 2/3 of the full screen (horizontal). The default display resolution was 1024 by 768 pixels.

Two regions of interest (ROI) corresponding to the speaker’s eyes and mouth were identified. The first being a 246 by 94 pixels rectangle that overlapped both eyes, while the second being a 163 (maximum horizontal length) by 100 (maximum vertical length) pixels ellipse that overlapped the mouth. The participants kept their head still on their chin and forehead rest approximately 70 cm away from the screen.

Two tones were used throughout the experiment, a dipping tone (tone 3) and a falling tone (tone 4). Acoustically, the dipping tone is the lengthiest and significantly longer than the shortest falling tone (43; 5). Both tones were presented using each of the four Mandarin vowels (a, e, i, u). For all vowels, the duration of lexical tones was calculated from the auditory onset to the auditory offset with Audacity software (2023). In the current study, two paired sample T-tests showed the auditory duration of Tone 3(M=817ms, SD=72.03) was significantly longer than that of Tone 4 (M=523ms, SD=80.99) (t (15) = 16.44, p<.001, Cohen’s d=4.11). Similarly, F0 of Tone 3(M= 124 Hz, SD=8.65) was significantly lower than that of Tone 4 (M=183Hz, SD=36.47) (t (15) = 6.72, p<.001, Cohen’s d=1.68) are significantly different.

Both speakers presented two versions of each tone and vowel combination (i.e., two different recordings of each combination). This resulted in 32 unique video recordings of each stimulus (2 speakers × 2 tones × 4 vowels × 2 versions). The corresponding video for each trial was clearly displayed, while the quality of the audio (listening condition) was manipulated to be one of three levels: clear (no distortion or noise), noisy (with background babble noise), or silent (no audio presented). Such three conditions created a gradient of auditory degrading in the experiment. The 32 video stimuli were presented in each listening condition twice, which led to a total of 192 trials in the experiment (64 in each listening condition).

### Procedure

At the start of each trial, a white fixation cross was displayed in the center of the screen over a black background for 500ms and stayed on the screen until a fixation on the cross was registered. A 200ms blank black screen then replaced the cross. Following which, a videoclip was presented laterally, left or right side on two thirds of the full screen. To avoid the fixation cross directing participant’s eye on the mouth region, the videoclips were presented laterally. This resulted in the viewing angle of the speaker’s face being 12 degrees (horizontal) by 15 degrees (vertical). Participants were required to identify the tone presented in the clip based on both the visual and audio cues and responded via the keyboard. The audio was presented using headphones at 70-75 dB. Participants responded to a dipping tone by pressing the Q button on the keyboard and responded to a falling tone by pressing the P button on the keyboard. The key press indicated the end of the current trial. Trials from all conditions were presented randomly in three blocks of 64 trials. In between each block, participants were allowed to take a break for as long as they wanted. The eye-movements of one eye were recorded using the Eyelink 1000 static eye-tracker (SR Limited) at 1000Hz, and the data was analysed offline using DataViewer (SR Research, Ottawa). Before each block of trials, a 9-point calibration was conducted.

## Results

Subject analysis is consistent with item analysis. We adopt the subject analysis result. The accuracy rate for each condition is presented in Table 1. A two-way repeated-measures ANOVA (listening conditions × language) showed the main effect of listening condition was significant, F (2,38) =185.66, p<.001, η2p =.91; response accuracy decreased as the auditory information was degraded to silence. There was no other significant main effect of language or interaction effect. For the silent condition, a one sample t-test was run to accuracy rates of Mandarin and English speakers against chance level (.50). English speakers performed significantly better than chance level (t (9) =2.95, p=.016, Cohen’s d=.93), but Mandarin speakers performed at chance level (t (10) =2.12, p=.060, Cohen’s d=.64).

**Table 1.** Accuracy for each condition

	Native Mandarin		Native English	
	Mean	SD	Mean	SD
Clear	0.98	0.02	0.96	0.05
Noisy	0.76	0.08	0.69	0.13
Silent	0.54	0.07	0.56	0.07

For the eye-tracking measures, the trial duration used in the analyses was defined as the duration from the onset of the video to the time when a response was given. Eye-tracking provides information on which parts of the speaker’s face a listener looks at when processing audiovisual stimuli, and whether these changes depend on task demands. For example, if the task requires more information to be gleaned from a specific visual cue (e.g., the mouth) one would expect longer gaze duration on such locations. Smaller numbers of overall fixations would also reflect how attention is focused on the location, as the listener would be moving their gaze around the visual field less frequently. As each syllable duration varies and the gaze durations on ROIs changed between ROIs with auditory information degraded, thus, eye-gaze duration (proportion of total trial time) was adopted and analysed along with the number of fixations at the two ROIs of mouth and eyes.

Furthermore, participants’ native language (Mandarin speaker vs. English speaker) was included as an additional factor for all the omnibus analyses; however, all interactions involving the two language groups were statistically non-significant (p > .05). As a result, participants from both groups were combined for the following reported analyses.

### Fixations

A two-way Analysis of Variance (ANOVAs) were conducted on the number of fixations with ROIs and listening condition (clear, noisy, and silent) being independent variables. Neither ROI nor listening condition main effect was statistically significant. The interaction effect of ROI and listening condition was significant (F (2, 40) = 5.85, p = .006, η2p =.23).

**Figure 2.**
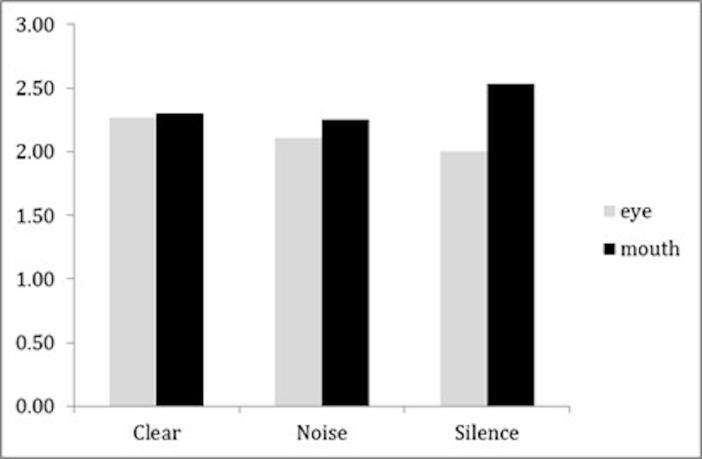
Mean number of fixations on each ROI.

Two repeated measures ANOVAs were conducted on the number of fixations for mouth and eyes respectively, with the listening condition being the independent variable. Figure 2 shows the number of fixations on each ROI in the different listening conditions. The difference in the number of fixations between each listening condition were statistically significant for both the eye ROI (F (2, 40) = 3.85, p = .030, η2p =.16) and the mouth ROI (F (2,40) = 5.55, p = .007, η2p =.22).

For the eye ROI, a paired sample T-test showed that participants did not show more fixations on the eye area during the clear condition (M=2.26, SD=1.79) compared to the silent condition (M=1.99, SD=1.50) (t (20) = 2.27, p = .102 with Bonferroni correction, Cohen’s d=.50). The same was true when examining differences between the noisy (M=2.10, SD=1.53) and silent conditions (t (20) = 1.78, p = .270 with Bonferroni, Cohen’s d=.389), as well as between the clear and noisy conditions (t (20) = 1.54, p = .417 with Bonferroni correction, Cohen’s d=.34).

For the mouth ROI, a paired sample T-test showed participants made significantly more fixations on the mouth area during the silent condition (M=2.53, SD=.99) compared to both the clear (M=2.30, SD=.90) (t (20) = 2.72, p = .039, Bonferroni correction, Cohen’s d=.594), and the noisy condition (M=2.26, SD=.93) (t (20) = 2.72, p =.039 with Bonferroni correction, Cohen’s d=.59). The difference in the number of fixations between the clear and noisy conditions was non-significant (t (20) = 0.45, p = .659 without Bonferroni correction, Cohen’s d=.10).

### Gaze duration

**Figure 3.**
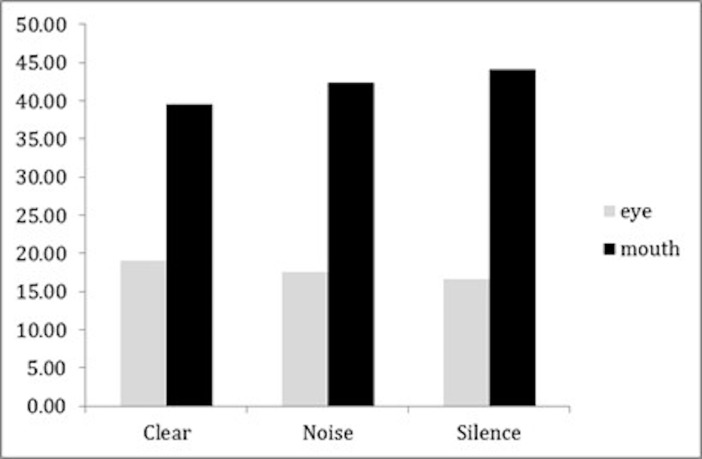
Percentage of gaze duration on each ROI.

A two-way repeated ANOVA (listening conditions × ROIs) was used to analyse gaze duration. Figure 3 shows the percentage of gaze duration on each ROI in the different listening conditions as a percentage of the entire duration of the trial. The main effect of ROI was statistically significant (F (2,40) = 20.04, p<.001, η2p =.30) and the interaction effect, with listening conditions, was also statistically significant (F (2, 40) = 8.41, p < .001, η2p =.50), but the main effect of listening condition was not significant. Simple effects analysis showed that ROI effect was statistically significant for all three listening conditions. It indicated all participants, both Chinese and English speakers, gazed longer at the mouth than eyes when perceiving lexical tones regardless of listening conditions.

Furthermore, the ROIs effect size at each listening condition was calculated as the percentages of gaze duration at the mouth subtracted with that of the eyes. The differences were reported as 20.5% (SE=.06) in clear condition, 24.7% (SE=.05) in noisy condition and 27.4% (SE=.05) in silence condition.

Paired sample T-tests were used to compare ROI differences between the two different auditory conditions. The result showed that ROI difference between clear and silent conditions was significantly different (t (20) = 3.42, p = .009 Bonferroni correction, Cohen’s d= .75). However, the difference of ROI between clear and noisy conditions was not significantly different (t (20) = 2.47, p = .069, Bonferroni correction, Cohen’s d= .54). The difference of ROI effect size between silent and noisy conditions was not significantly different (t (20) = 2.097, p = .147, Bonferroni correction, Cohen’s d= .46).

## Discussion

The current study revealed that both Chinese and English speakers would turn to the mouth area rather than eyes area when processing lexical tones, in both clear and adverse conditions, but particularly in the silent condition. It suggests that the mouth becomes relevant whenever visual support is needed for speech. This finding supported the primacy of the mouth in the perception of visual tones (25). Moreover, the similar eye movement patterns between the Chinese and English speakers implies that, regardless of native language, the mouth area acts as a cue for perceptual processing of lexical tones. In contrast, the role of eyes or upper facial areas are assumed to facilitate perceptions of intonation and convey social or pragmatic information. In addition, compared to gaze fixations, gaze duration appeared a more sensitive indicator of how listeners allocated their gaze when perceiving audiovisual tones gaze duration not only revealed the primacy of mouth, but it also increased as listening conditions became adverse. On the contrary, the number of fixations only increased at mouth area in silent condition. The role of the gaze at the mouth and the eyes is an important issue in many audiovisual speech studies (38; 12; 3; 44; 39; 47). The issue is predominantly focused on two questions raised from the current findings.

Firstly, why would the mouth be more relevant to the processing of lexical tones? It is assumed that the mouth region provides articulatory cues that are relevant to the different speech units, e.g., consonants and vowels. Few studies have suggested the mouth to be relevant to lexical tone production. Most have investigated segmental information, but at least one has looked into prosodic information, i.e., stress. Like lexical tone, word’s stress pattern is borne by a relatively short syllable. Cruz et al. (9) reported that eye gaze to the mouth region was modulated by stress pattern in disyllable. Infants who did not show an iambic preference paid more attention to the mouth. As lexical tone is also carried on syllabic level, it might be the case that looking at the mouth generally helps with lexical tone processing when hearing a relatively short syllable or vowel (especially in situations where subjects are struggling with processing speech).

The current study supported the primacy of the mouth in audiovisual lexical tone perception. Studies on visual benefits have confirmed the existence of visual cues to facilitate perceptions of lexical tone. Compared to the mouth, the eyes provide little information relating to the production of speech, yet have been demonstrated to provide pragmatic information, which is generally borne and conveyed through intonation. If lexical tone is processed perceptually and borne by vowels, then the mouth may be more useful than the eyes or upper facial area.

However, the primacy of mouth did not offer a transparent link between the visual cues from the mouth and the lexical pitch contours. Indeed, no study has addressed how such gaze would provide a specific visual cue relevant to the perceptual targets. For example, Vatikiotis-Bateson et al (41) did not find any correlation between phoneme identification performance and eye-movement. Paré, Richler, ten Hove and Mundell (32) confirmed that in audiovisual speech perception, participants’ gaze primarily focused on the mouth and the eye regions. However, these gaze fixations did not predict the likelihood of the McGurk effect occurring, which indexed perceptual confusion occurring at the segment-level.

In future studies, an eye-tracker device could be used to measure the eye movement patterns associated with visual timing information. For instance, the visual duration of lexical tone. Summerfield (36) claimed that timing information was defined as the duration between the onset and offset of the segment. Best, Ozmera, and Shinn-Cummingham (2) showed that visual timing information could improve identification accuracy. Xie, Zeng and Wang (42) reported preliminary results that suggest lip movement duration, one form of visual timing cues, could facilitate the discrimination of Mandarin lexical tones. In this sense, visual timing information would cue the participants attention towards the auditory stimulus.

Secondly, why doesn’t native language emerge as a relevant factor? Previous studies on audiovisual speech perception demonstrated how native English speakers were significantly more affected by visual cues compared to native Mandarin speakers when they listened to audiovisual syllables. Such difference was once interpreted as not only due to the cultural and language background but also to the phonetic and acoustic characteristics of the specific language and speaker’s visual appearance of the stimuli. However, recent studies (27) have revealed both English and Chinese speakers showed similar McGurk ratios when processing audiovisual stimuli. Specifically, Burnham et al. (5) found that visual augmentation of auditory tone perception in noise was evident for tonal and non-tonal language groups. The current study contributes evidence from Mandarin lexical tones. Results revealed that, after some brief training, native English speakers performed comparably with native Mandarin speakers when identifying lexical tones. This suggests audiovisual integration might be a universal sensory ability, allowing listeners to even detect lexical tones in their non-native languages.

Both Mandarin and English speakers showed similar eye gaze patterns when processing audiovisual lexical tones. This absence of difference between the two groups might be due to phonetic level processing only. This poses the question of whether audiovisual processing of lexical tones is more reliant on visual phonetic cues or visual phonological cues. The processing level of lexical tones has long been a controversial topic (48; 35). For auditory processing, it is acknowledged that non-tonal language speakers, e.g., English speakers, are capable of identifying lexical tones. However, we do not have enough information to know the extent to which tone is being processed as a category by Mandarin speakers, and whether the F0 contours are processed as pitch categories by English speakers. In other words, how do we know if speakers are processing the audiovisual signal at the phonetic level or at the phonological level? This question is worthy of further investigation.

### Ethics and Conflict of Interest

The author(s) declare(s) that the contents of the article are in agreement with the ethics described in http://biblio.unibe.ch/portale/elibrary/BOP/jemr/ethics.html and that there is no conflict of interest regarding the publication of this paper.

### Acknowledgements

The study was funded by the British Academy Small Grant (SG152162). Two anonymous reviewers are thanked for critically reading the manuscript and suggesting substantial improvements. We also acknowledge Dr Rui Wang in data collection and Ms. Bridie Stone and Mr. Josh Molina for proofreading.
